# Quantitative proteomics analysis in small cell carcinoma of cervix reveals novel therapeutic targets

**DOI:** 10.1186/s12014-023-09408-x

**Published:** 2023-04-08

**Authors:** Haifeng Qiu, Ning Su, Jing Wang, Shuping Yan, Jing Li

**Affiliations:** 1 Department of Gynecology, The First Affiliated Hospital of Zhengzhou University, Zhengzhou, Henan China; 2 Provincial Medical Key Laboratory for Gynecologic Malignancies Prevention and Treatment, Zhengzhou, Henan China; 3 Zhengzhou Key Laboratory for Gynecologic Malignancies Prevention and Treatment, Zhengzhou, Henan China; 4 Department of Gynecologic Oncology, The Affiliated Cancer Hospital of Zhengzhou University and Henan Cancer Hospital, Zhengzhou, Henan China; 5 Department of Obstetrics and Gynecology, The Affiliated Yantai Yuhuangding Hospital of Qingdao University, Yantai, Shandong China; 6 Department of Pathology, The First Affiliated Hospital of Zhengzhou University, Zhengzhou, Henan China; 7 Department of Oncology, The First Affiliated Hospital of Zhengzhou University, No.1, East Jianshe Road, Erqi District, Zhengzhou, 450000 Henan China

**Keywords:** Small cell carcinoma of the cervix, Quantitative proteomics analysis, DNA replication, Cellular motility, Metabolism, Therapeutic targets

## Abstract

**Background:**

As a rare pathologic subtype, small cell carcinoma of the cervix (SCCC) is characterized by extensive aggressiveness and resistance to current therapies. To date, our knowledge of SCCC origin and progression is limited and sometimes even controversial. Herein, we explored the whole-protein expression profiles in a panel of SCCC cases, aiming to provide more evidence for the precise diagnosis and targeting therapy.

**Methods:**

Eighteen SCCC samples and six matched normal cervix tissues were collected from January 2013 to December 2017. Data independent acquisition mass spectrometry (DIA) was performed to discriminate the different proteins (DEPs) associated with SCCC. The expression of CDN2A and SYP in corresponding SCCC tissues was verified using immunohistochemistry. GO and KEGG enrichment analyses were used to identify the key DEPs related to SCCC development and tumor recurrence.

**Results:**

As a result, 1311 DEPs were identified in SCCC tissues (780 up-regulated and 531 down-regulated). In up-regulated DEPs, both GO analysis and KEGG analysis showed the most enriched were related to DNA replication (including nuclear DNA replication, DNA-dependent DNA replication, and cell cycle DNA replication), indicating the prosperous proliferation in SCCC. As for the down-regulated DEPs, GO analysis showed that the most enriched functions were associated with extracellular matrix collagen-containing extracellular matrix. KEGG analysis revealed that the DEPs were enriched in Complement and coagulation cascades, proteoglycans in cancer, and focal adhesion-related pathways. Down-regulation of these proteins could enhance the mobility of cancer cells and establish a favorable microenvironment for tumor metastasis, which might be accounted for the frequent local and distant metastasis in SCCC. Surprisingly, the blood vessels and circulatory system exhibit a down-regulation in SCCC, which might be partly responsible for its resistance to anti-angiogenic regimens. In the stratification analysis of early-stage tumors, a group of enzymes involved in the cancer metabolism was discriminated in these recurrence cases.

**Conclusions:**

Using quantitative proteomics analysis, we first reported the whole-protein expression profiles in SCCC. Significant alterations were found in proteins associated with the enhancement of DNA replication and cellular motility. Besides the association with mitosis, a unique metabolic feature was detected in cases with tumor recurrence. These findings provided novel targets for disease surveillance and treatments, which warranted further validation in the future.

**Supplementary Information:**

The online version contains supplementary material available at 10.1186/s12014-023-09408-x.

## Background

Small cell carcinoma of the cervix (SCCC) is a relatively rare subtype that accounts for < 1% of all the malignancies originating from the cervix [1, 2]. However, the extensive aggressiveness and multiple resistance to current regimens made it the most lethal cervical cancer [3]. In the United States, the 5-year survival rates of SCCC were 81.8% (stage IA), 55.4% (stage IB), 22.2% (stage IIB), 24.4% (stage IIIB), 4.1% (stage IVA), and 7.1% (stage IVB). These were much poorer than the outcomes of patients with either squamous cancer or adenocarcinoma [4]. Similar findings were also reported by Zheng et al. In a Chinese SCCC cohort, the 3-year survival rates were 100% (stage IA), 62% (stage IB1), 53% (stage IB2), 36% (stage IIA), 29% (stage IIB), 50% (stage IIIB), and 0% (stage IVA) [5]. Due to its rarity, the current publications about SCCC are mostly descriptive and based on small groups, which results in low-quality data and even controversies. For example, in a study containing 188 SCCCs, the authors concluded that adjuvant chemo- or chemoradiotherapy was associated with improved survival in patients with stages IIB-IVA tumors [6]. Furthermore, Wei et al. also reported that radiotherapy could improve the prognosis of SCCC, regardless of tumor stage [7]. Consistently, brachytherapy was associated with improved overall survival in locally advanced small cell carcinoma of the cervix (SCCC), which was underutilized in clinical practice [8]. However, in a meta-analysis that included the information of 1,904 SCCC patients, the authors found that adjuvant radiotherapy might not be helpful to improve the treatment outcomes [9]. Moreover, the follow-up of 68 Korean SCCC patients also demonstrated that a combination of chemotherapy and radiation showed no more benefits than single chemotherapy. In addition, the authors claimed that patients who received neoadjuvant chemotherapy showed a poorer prognosis than those who did not [10]. In a Chinese cohort containing 93 SCCC patients, Li et al. found that the FIGO stage was the only prognostic factor, while treatment modality did not have an impact on overall survival [11]. These different findings were largely attributed to our limited knowledge about SCCC, which significantly hindered the precise prevention and targeting therapy. Thus, further exploring the underlying molecular mechanisms in SCCC is urgently needed.

Several studies have reported the genetic alterations in SCCC. In a panel of eight SCCC cases, the common genetic events were LOH at 9p21 (42.9%) and 3p deletions (37.5%). One study found that mutations of the *P53* gene occurred in 62.5% of patients while no K-Ras mutation was detected [12]. However, a later study proved that both LOH and *P53* mutations were rare events in 10 SCCC patients [13]. In 2016, Lee et al. performed whole-exome sequencing to investigate the integrative mutation profiles of SCCC. They reported the most frequent mutations were found in *ATRX*, *ERBB4*, *PTEN*, *RICTOR*, and *TSC1/2* genes, implying their potential functions during the initiation and development of SCCC [14]. In a recent study, the authors investigated the mutations in 10 SCCC cases using a next-generation sequencing-based 637-gene panel. The common mutations were detected in *P53* (40%) and *PIK3CA* (30%). Rare mutations were also detected in *K-RAS*, *c-MYC*, *NOTCH1*, *BCL6* or *NCOA3*, *PTEN*, *RB1*, *BRCA1*, *BRCA2*, and *ARID1B* [15].

Besides the alterations at DNA and RNA levels, the alterations of protein expression and activity also showed significant influences in cancers. Quantitative proteomics is a novel concept for systematically analyzing the whole-protein profiles in a cell, tissue, organ, or other biological systems [16, 17]. It can decipher not only aberrations of protein expression but also post-translational modifications that affect the functions of certain proteins [18, 19]. In the present study, we performed a quantitative proteomic analysis in a panel of SCCC patients, aiming to illustrate the key proteins and pathways involved in this lethal malignancy.

## Materials and methods

### Sample collection

A total of 18 SCCC and six matched normal cervix (at least 2 cm away from tumor margin) FFPE tissues were collected in the Department of Gynecology, First Affiliated Hospital of Zhengzhou University from January 2013 to December 2017. H&E and IHC slides were re-evaluated by experienced pathologists to confirm the diagnosis. The clinic-pathological information was extracted from medical records and summarized in Table 1. Tumor stages were determined according to FIGO criteria (version 2018). The detection of 21 HPV subtypes was performed using the assay kit obtained from Hybribio (Hong Kong, China). Follow-up was performed face-to-face in the outpatient office or by telephone. Overall survival (OS) was defined as the period from the diagnosis to the death. Disease-free survival (DFS) was defined as the period from the time of surgery to the diagnosis of tumor recurrence/metastasis. Formal consents were provided by all patients and our study was approved by the Ethical Committee at the First Affiliated Hospital of Zhengzhou University.

**Table 1:** The baseline characteristics of SCCC patients

Case	Age	HPV	Stage	Tumor size(cm)	Invasion depth	LVSI	LNM	DFS	OS	Prognosis
Ca001	51	18	IB3	4.5 × 3	> 4/5	Negative	Negative	13	23	DOD
Ca002	38	16	IIIC1p	6 × 5	> 4/5	Positive	Positive	31	41	DOD
Ca003	26	18	IIA2	5 × 3.5	2/3	Positive	Negative	8	13	DOD
Ca004	37	18/58	IIIC1p	4 × 2.5	2/3	Positive	Positive	8	17	DOD
Ca006	58	18/51	IB2	3.5 × 3.3	1/2	Negative	Negative	66	66	Alive
Ca007	60	Negative	IIIC1p	3.4 × 4.9	> 4/5	Negative	Positive	11	16	DOD
Ca009	64	ND	IIA1	1 × 1	< 1/3	Negative	Negative	19	31	DOD
Ca054	44	16/18	IIIC1p	3 × 3.5	3/4	Positive	Positive	22	27	DOD
Ca055	50	18/39	IB1	1.3 × 1	< 1/3	Negative	Negative	10	15	DOD
Ca056	49	18	IIA1	3.5 × 2.4	> 4/5	Negative	Negative	45	57	AWD
Ca057	29	18/45	IIIC1p	4.5 × 3.5	> 4/5	Positive	Positive	22	39	DOD
Ca058	47	Negative	IB2	1.9 × 2.5	< 1/3	Negative	Negative	62	62	Alive
Ca059	52	16	IB1	1.5 × 1	> 4/5	Positive	Negative	13	16	DOD
Ca060	40	18	IB3	5.5 × 4	1/2	Negative	Negative	8	26	DOD
Ca061	44	Negative	IIIC1p	2.2 × 2	> 4/5	Positive	Positive	29	48	DOD
Ca252	38	ND	IIB	2.8 × 2.0	> 4/5	Positive	Negative	6	30	DOD
Ca253	30	18/52/66	IIIC1p	1.2 × 1.6	> 1/2	Positive	Positive	7	15	DOD
Ca254	48	33/51	IB2	2.3 × 1.7	> 4/5	Positive	Negative	18	27	DOD

ND: not detected; LVSI: lymphovascular invasion; LNM: lympho node metastasis; DFS: disease-free survival; OS: overall survival; DOD: dead of disease; AWD: alive with recurrent disease

### Protein extraction, quality control

Total protein was extracted from FFPE tissue with 10 μm thickness. Briefly, after dewaxing and rehydration, ~ 1 cm^2^ FFPE tissue sections were thoroughly mixed with 200 μl lysis buffer (4% SDS, 1% protease inhibitor cocktail (Sigma) and put on ice for 15 min. Then the samples were ultrasonicated for 10 min and then heated at 95 °C, 750 rpm for 1 h. After centrifugation, supernatants were collected and a BCA assay was performed to determine the protein concentration.

### Sample enzymolysis and desaltation

The samples were treated with DTT solution (2 µL 500 mM) in a water bath at 56 °C for 1 h. Then 20 µL IAM (500 mM) was added and the samples were kept at room temperature blocked from light for 45 min. 60 µg protein was purified with SP3 beads (single-pot solid-phase-enhanced sample preparation, GE Healthcare) and a 20 µL digestion buffer (50 mM NH_4_CO_3_, 50 mM CaCl_2_, 2.4 µg trypsin) was used to resuspend the beads. The proteins were digested overnight at 37 °C and 1 µg trypsin was further added to extend the digestion for another 3 h at 37 °C. Then the resulting peptides was precipitated on the beads using final concentration of 95% ACN, and washed with 100% ACN. Peptides were eluted from SP3 beads by 2% ACN.

### MS database generation

A total of 100 µg of digested peptides that mixed equally volume from different samples was pre-separated with Waters XBridge Shield C18 RP column, 3.5 um, 4.6 × 250 mm on HPLC Shimadzu LC20AD (Shimadzu, Japan) with a 90 min gradation. Mobile phases A (H_2_O, adjusted pH to 10.0 using ammonium hydroxide) and B (80% acetonitrile) were used to develop a gradient elution. The solvent gradient was set as follows: 0–5 min, 5%B; 5–25 min, 5–12%B; 25–60 min, 12–22%B; 60–70 min, 22–35%B; 70–75 min, 35–80%B; 75–80 min, 80%B; 80–82 min, 80–5%B, 82–90 min, 5%B, and 1 mL/min flowrate. The eluates were collected for a tube per minute and merged into 10 fractions. All fractions were dried under vacuum and reconstituted in 2% ACN in water. After mix with 0.2 µL standard peptides, the fraction samples were used for subsequent analyses.

Transition library construction was performed using an Ultimate RSLC nano 3000 UHPLC system coupled with an Orbitrap Q Exactive HF mass spectrometer (Thermo Fisher) operating in data-dependent acquisition (DDA) mode. Each fraction sample containing iRT was injected into a Thermo Acclaim PepMap RSLC C18 column (2 µm, 75 µm × 75 cm) and analyzed with gradient elution as follows ( A:2% ACN, 0.1% FA; B:80% ACN, 0.1% FA): 0–8 min, 3–6% B; 8–9 min, 6% B; 9–30 min, 6–12% B;30–105 min, 12–24% B; 105–125 min, 24–35% B; 125–126 min, 35–90% B; 126–141 min, 90% B; 141–142 min, 6% B; 142–150 min, 6% B. The Q-Exactive HF mass spectrometer was operated in positive polarity mode with spray voltage of 2.0 kV and capillary temperature of 250 °C. Full MS scans range from 350 to 1800 m/z were acquired at a resolution of 60,000 (at 200 m/z) with a gain control (AGC) target value of 1 × 10^6^ and a maximum ion injection time of 60 ms. The top 40 most abundant precursor ions from MS1 were selected for fragmentation using higher energy collisional dissociation (HCD). The fragment ions were analyzed in MS2 at a resolution of 30,000 (at 200 m/z) with the conditions as follows: AGC target value was 1 × 10^5^, the maximum ion injection time was 90 ms, a normalized collision energy of 29% and the dynamic exclusion parameter of 9s.

### DIA data acquisition

Peptides of each sample was reconstituted in mobile phases A (2% ACN, 0.1% FA) and mixed with 0.2 μL 10 × iRT standards (Biognosys, Schlieren, Switzerland). The data of lysed peptide samples were acquired through an Orbitrap Q Exactive HF mass spectrometry (Thermo Fisher Scientific, Waltham, MA) in the data-independent acquisition (DIA) mode with spray voltage of 2.0 kV, Nanospray Flex™ (ESI) and capillary temperature of 250 °C. For DIA acquisition, the m/z range covered from 350 to 1800 m/z with 40 scan windows. The liquid conditions were the same as described above. The MS1 resolution was set to 60,000 (at 200 m/z) with the full scan AGC target value of 1 × 10^6^ and the maximum ion injection time of 50 ms. Peptides were fragmented by HCD with aa normalized collision energy of 29% and detected in MS2 with the resolution of 30,000 (at 200 m/z) and AGC target value of 1 × 10^6^.

### Data analysis

The DIA data were searched against the human UniProt database (20,365 sequences) using Spectronaut (v14.5.200813.47784). The library generation with DDA data applied the default settings with trypsin/P digest rule. 2 missed trypsin cleavage was allowed by default. The precursor peptide mass tolerance was 20 ppm, and fragment ion mass tolerance was 0.02 Da. The FDR cutoff for precursor and protein identification was 0.01, and other parameters were set as default.

### Validation of CDN2A and SYP expressions using immunohistochemistry (IHC)

First, 5 μm slides were dewaxed and re-hydrated, and antigen retrieval was done in a microwave. Then the endogenous peroxidase activity was blocked and samples were incubated with primary antibodies at 4 °C overnight. On the second day, the slides were incubated with secondary antibodies for 30 min at 37 °C. Next, the specific staining was developed using a commercial kit (Zhong Shan-Golden Bridge Biological Technology, Beijing, China). The staining results were evaluated by two pathologists in our hospital.

### Quantification and statistical analysis

The output quantified proteins with at least 30% appearance in all samples were selected for further analysis. The proteins were normalized with the FOT (Fraction of Total) method that normalized every protein intensity equals to every-protein-intensity/all-protein-intensity-in-one-sample × 1,000,000. The absent values were replaced by the half of the minimum value in the original data. FC (Fold change) ≥ 2 and *P* < 0.05 were set as the cut-off for differential proteins (DEPs). It should be noted that we adjusted a relatively loose thresholds with FC ≥ 1.5 and *P* < 0.05 as the cut-off for DEPs in comparison of recurrent and non- recurrent group to gain the maximum information from limited samples. The GO and KEGG functional gene enrichments were achieved through the R package (clusterProfiler, v3.16.1). The annotation database was org.Hs.eg.db (v3.11.4). The background proteins were set with all the quantified proteins. The differential proteins were input to generate the enrichment pathway list and figures. All the data was deposited in the Integrated Proteome Resources (Project number: PXD035382).

## Results

### Baseline characteristics of SCCC patients

Considering its rarity, we screened all patients diagnosed with cervical cancer in our hospital from January 2013 to December 2017. In total, there were 3,092 squamous cell carcinoma (87.0%), 317 adenocarcinoma (8.9%), 25 SCCC (0.7%) and 121 other subtypes (3.4%). After re-evaluation, 18 SCCC cases with intact clinic-pathological records were qualified for the following analysis, including 7 stage IB (2 IB1, 4 IB2, 1 IB3), 4 stages II (2 IIA1, 1 IIA2, 1 IIB), and 7 stage IIIC1p tumors. The median age was 44 years old (ranging from 26 to 64). In 16 patients who had taken a high-risk HPV test, HPV18 was the most common subtype (62.5%, 10/16), while three patients were high-risk HPV-negative (18.8%). The details of patients’ characteristics were listed in Table 1.

### Overview of the proteomics profiles

**Fig. 1:**
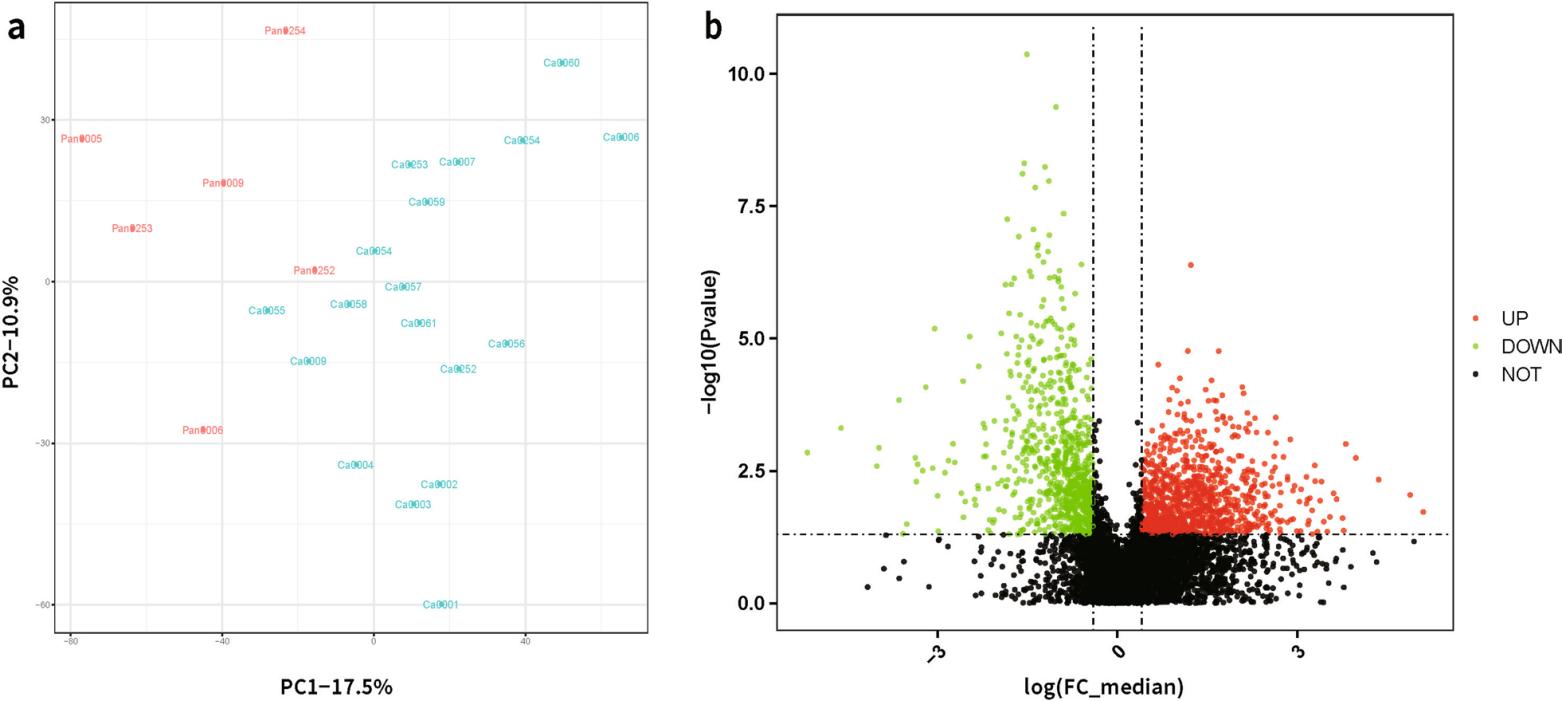
PCA analysis and volcano plot. **a** PCA analysis indicated a clear separation between SCCC (blue, title with Ca + sample number) from the normal cervix samples (red, titled with Pan + sample number). **b** The X axis represents fold changes of proteins (log_2_FC), and the Y axis the indicated the corresponding P values. The up- and down-regulated proteins were indicated with green and red dots. The gray dots meant no significant changes of these proteins

Using the conventional DDA mass spectrometry, we established a protein library of the normal human cervix (n = 6) and SCCC (n = 18) tissues. The DIA-MS data was searched against the DDA library and a total of 7,819 proteins were identified. After being filtered, 6786 proteins were quantified in at least 30% samples and the missing values were replaced by the half of the minimum value in the original data (Additional file 1: Table S1). To assess the reliability and reasonability of our experiment, a Pearson correlation and Principal Component Analysis (PCA) were calculated using all proteins retained. All the results indicated a clear separation between SCCC from the normal cervix (Fig. 1a).

### Changes in protein expression in SCCC

To examine the abnormalities in SCCC, differentially expressed proteins between SCCC and normal cervix were defined with the cut-off of FC ≥ 2 and *P* < 0.05. As a result, 1311 proteins were differentially expressed in SCCC with 780 up-regulated and 531 down-regulated compared with normal control (Fig. 1b and Additional file 2: Table S2). The top-30 DEPs and their details were listed in Tables 2 and 3.

**Table 2:** Top 30 up-regulated DEPs

Accessions ID	Genes	Protein descriptions(Full name)	PG.Protein names	log2_FC_median (log ratio)	FC_median (ratio)	Pvalue	Ajusted pvalue	median_normal	median_SCCC	Avg (Normal)	Avg (SCCC)	Std (Normal)	Std (Normal)
Q9UEW8	STK39	STE20/SPS1-related proline-alanine-rich protein kinase	STK39_HUMAN	7.36119336	164.4144581	0.018860878	0.101659984	211.4552765	34,766.30469	1527.338822	49,956.00369	2848.88591	44,771.05061
Q9Y3R5	DOP1B	Protein dopey-2	DOP2_HUMAN	7.047271208	132.2635031	0.009017667	0.067617556	390.7667237	51,684.17579	455.894511	53,527.82245	145.6301596	43,456.97949
Q9UBW7	ZMYM2	Zinc finger MYM-type protein 2	ZMYM2_HUMAN	6.288898031	78.18923194	0.004630235	0.050678673	529.169983	41,375.39454	4620.847708	40,939.30145	9149.269534	26,504.5089
P12532	CKMT1A	Creatine kinase U-type, mitochondrial	KCRU_HUMAN	5.738345477	53.38436902	0.001805234	0.030247698	16,038.71924	856,216.9063	25,622.14437	917,631.895	18,427.92428	589,576.229
P42771	CDKN2A	Cyclin-dependent kinase inhibitor 2A	CDN2A_HUMAN	5.498126381	45.19609998	0.000982386	0.021234095	4311.488404	194,862.461	7345.865885	220,803.6349	8252.474718	131,670.3183
O75146	HIP1R	Huntingtin-interacting protein 1-related protein	HIP1R_HUMAN	5.44650024	43.60737491	0.042449977	0.159768091	556.9887085	24,288.81543	4073.540395	23,434.23526	7863.248618	20,583.82942
Q9BVW5	TIPIN	TIMELESS-interacting protein	TIPIN_HUMAN	5.420841786	42.83867003	0.024706829	0.120254705	1097.918701	47,033.37696	1097.918701	53,906.45801	2.27E-13	51,368.51886
Q9H6K5	PRR36	Proline-rich protein 36	PRR36_HUMAN	5.277000873	38.77354861	0.010817348	0.075164197	1270.526734	49,262.83008	1482.28119	44,692.1954	473.497357	36,396.36268
Q96GM5	SMARCD1	SWI/SNF-related matrix-associated actin-dependent regulator of chromatin subfamily D member 1	SMRD1_HUMAN	5.204188829	36.86522939	0.008387325	0.065647507	1221.024536	45,013.34961	6056.397216	53,136.54706	7348.723373	37,892.81275
P11388	TOP2A	DNA topoisomerase 2-alpha	TOP2A_HUMAN	5.115361941	34.66389679	0.023438944	0.116781697	1477.363159	51,211.16407	1946.478332	83,853.67923	1048.973417	78,872.81998
Q460N5	PARP14	Protein mono-ADP-ribosyltransferase PARP14	PAR14_HUMAN	5.051141315	33.15469584	0.044442	0.163637227	9322.803161	309,094.7032	113,391.6547	370,861.0882	178,506.0581	263,836.9669
Q8NFP9	NBEA	Neurobeachin	NBEA_HUMAN	4.967745586	31.29251235	0.028754464	0.130171978	1074.147766	33,612.78223	5972.771382	43,643.51539	7428.877063	37,508.36122
P12314	FCGR1A	High affinity immunoglobulin gamma Fc receptor I	FCGR1_HUMAN	4.913751049	30.14299885	0.005041107	0.052147793	2469.903321	74,450.29297	2881.553874	99,400.99513	920.4786202	72,657.58303
P52756	RBM5	RNA-binding protein 5	RBM5_HUMAN	4.878839207	29.42232208	0.011560853	0.077216487	2238.103272	65,850.19532	19,502.76416	67,707.54153	25,939.69471	38,207.43809
P0DN79	CBSL	Cystathionine beta-synthase-like protein;Cystathionine beta-synthase	CBSL_HUMAN;CBS_HUMAN	4.814433533	28.13772013	0.044546292	0.163843433	2436.617432	68,560.85938	2842.720337	88,476.12218	908.0737018	94,257.57237
P18858	LIG1	DNA ligase 1	DNLI1_HUMAN	4.761255341	27.11943732	0.004953393	0.051713424	4643.395508	125,926.2735	6351.933756	133,251.627	2432.224844	95,289.99818
P40938	RFC3	Replication factor C subunit 3	RFC3_HUMAN	4.751387685	26.9345804	0.002498748	0.036112277	1982.11731	53,387.49805	12,027.9799	66,241.66961	14,209.03093	36,349.16249
Q96S86	HAPLN3	Hyaluronan and proteoglycan link protein 3	HPLN3_HUMAN	4.687638778	25.77032414	0.049269072	0.1739184	3618.05542	93,238.46093	13,298.16699	153,378.2993	20,717.3008	157,400.3024
O14683	TP53I11	Tumor protein p53-inducible protein 11	P5I11_HUMAN	4.686881021	25.75679216	0.017581321	0.097509494	1945.32251	50,105.26758	2269.542928	62,968.96597	724.9788951	55,452.83062
Q6NXG1	ESRP1	Epithelial splicing regulatory protein 1	ESRP1_HUMAN	4.632471609	24.80349659	0.010347592	0.07323217	2582.032471	64,043.4336	17,814.92733	63,677.24805	22,823.02529	36,027.97701
P09326	CD48	CD48 antigen	CD48_HUMAN	4.608239287	24.39036236	0.027588616	0.126754466	1614.560547	39,379.7168	4874.363201	52,028.30914	5500.379072	46,760.21024
Q16576	RBBP7	Histone-binding protein RBBP7	RBBP7_HUMAN	4.58773499	24.04616619	0.032354519	0.13858515	2554.079346	61,415.81641	7752.206951	68,417.96881	10,522.55828	61,982.56732
Q8NBJ4	GOLM1	Golgi membrane protein 1	GOLM1_HUMAN	4.575636972	23.84536517	0.010995783	0.075431856	2863.917237	68,291.15235	17,917.06983	69,412.97833	21,812.35588	41,626.55051
Q99436	PSMB7	Proteasome subunit beta type-7	PSB7_HUMAN	4.551344635	23.44721447	0.013162013	0.083884171	9781.66699	229,352.8438	43,456.63834	255,166.8022	71,014.90995	179,459.6422
Q9NVP2	ASF1B	Histone chaperone ASF1B	ASF1B_HUMAN	4.550893393	23.43988185	0.004111323	0.046576686	4228.997559	99,127.20313	4228.997559	117,532.7544	0	82,982.34381
Q9H5H4	ZNF768	Zinc finger protein 768	ZN768_HUMAN	4.421533747	21.42961086	0.007044493	0.060974403	1769.069214	37,910.46485	1769.069214	33,820.35429	2.27E-13	25,296.06673
Q9NX00	TMEM160	Transmembrane protein 160	TM160_HUMAN	4.409021661	21.2445615	0.04356989	0.16138934	88,124.80078	1,872,172.75	649,803.9105	1,881,539.407	844,979.995	1,257,655.391
Q9Y6V7	DDX49	Probable ATP-dependent RNA helicase DDX49	DDX49_HUMAN	4.378939624	20.8061716	0.012018926	0.079416196	491.5249024	10,226.75146	491.5249024	11,117.62381	0	9103.12609
O60725	ICMT	Protein-S-isoprenylcysteine O-methyltransferase	ICMT_HUMAN	4.319314494	19.9638006	0.043375703	0.161197986	2481.373047	49,537.63672	2894.935222	66,127.60634	924.7531351	69,178.19749
Q96P11	NSUN5	Probable 28S rRNA (cytosine-C(5))-methyltransferase	NSUN5_HUMAN	4.265183131	19.22861745	0.006070132	0.057450371	1004.54303	19,315.97364	2422.19635	21,998.87501	2745.33897	15,040.52151

**Table 3:** Top 30 down-regulated DEPs

Accession ID	Gene name	PG.Protein descriptions(Full name)	PG.Protein names	log2_FC_median (log ratio)	FC_median (ratio)	P value	Ajusted pvalue	median_normal	median_SCCC	Avg (Normal)	Avg (SCCC)	Std (Normal)	Std (Normal)
O60262	GNG7	Guanine nucleotide-binding protein G(I)/G(S)/G(O) subunit gamma-7	GBG7_HUMAN	-7.465063811	0.00565968	0.001427786	0.026242633	187,473.2032	1061.03833	228,984.7346	25,799.27938	212,626.8755	44,948.5899
P06401	PGR	Progesterone receptor	PRGR_HUMAN	-6.65413918	0.009928977	0.000491759	0.014831463	81,027.86329	804.5238035	105,418.7344	12,755.5307	86,412.66179	18,514.38528
Q8N135	LGI4	Leucine-rich repeat LGI family member 4	LGI4_HUMAN	-5.791082391	0.018059698	0.0025649	0.036565985	52,110.63868	941.1024173	78,544.74316	13,855.35018	64,985.93539	24,125.71286
O00292	LEFTY2	Left–right determination factor 2	LFTY2_HUMAN	-5.740812741	0.018700067	0.00116171	0.023251825	115,278.7813	2155.720948	107,676.8767	17,601.85626	79,873.06062	32,868.47761
P46439	GSTM5	Glutathione S-transferase Mu 5	GSTM5_HUMAN	-5.25872318	0.026119604	0.000145872	0.00649374	102,030.8594	2665.005615	105,055.2268	13,949.63367	72,982.40506	19,991.41695
Q13077	TRAF1	TNF receptor-associated factor 1	TRAF1_HUMAN	-5.164490606	0.027882609	0.048823649	0.173102028	91,780.00963	2559.066162	2,002,717.525	116,916.7609	3,645,519.056	258,177.4092
O95969	SCGB1D2	Secretoglobin family 1D member 2	SG1D2_HUMAN	-5.072841905	0.029711353	0.031939638	0.137526893	116,090.9941	3449.220459	166,550.759	39,330.52087	181,847.1032	77,095.79326
O95428	PAPLN	Papilin	PPN_HUMAN	-4.867594077	0.034253754	0.001794389	0.030215193	48,590.07422	1664.392456	40,443.21847	8079.124825	30,260.2273	12,323.00884
Q6PEW1	ZCCHC12	Zinc finger CCHC domain-containing protein 12	ZCH12_HUMAN	-4.841581438	0.034876971	0.005062549	0.05228989	55,883.96289	1949.063355	89,396.37985	7743.709066	105,339.8753	9158.160269
Q13268	DHRS2	Dehydrogenase/reductase SDR family member 2, mitochondrial	DHRS2_HUMAN	-4.809502642	0.035661158	0.002372941	0.035158908	174,346.1133	6217.384278	225,498.5329	34,636.61866	210,831.0987	46,664.27529
Q13444	ADAM15	Disintegrin and metalloproteinase domain-containing protein 15	ADA15_HUMAN	-4.68794636	0.038796052	0.003131617	0.040172306	51,262.61329	1988.786988	80,436.83295	13,465.98901	76,357.98991	17,270.21737
P07101	TH	Tyrosine 3-monooxygenase	TY3H_HUMAN	-4.614625852	0.040818703	8.30496E-05	0.004534772	86,297.32032	3522.544678	83,258.55396	6597.327678	64,171.75878	4795.18235
Q00604	NDP	Norrin	NDP_HUMAN	-4.446923869	0.045850336	0.002824128	0.038176363	142,096.1172	6515.154785	298,987.4484	9634.858996	349,533.5564	6932.883615
Q6ZRY4	RBPMS2	RNA-binding protein with multiple splicing 2	RBPS2_HUMAN	-4.400859229	0.047337941	6.53305E-06	0.00090476	73,665.48438	3487.172364	62,417.65707	6789.351861	37,087.85008	5887.241426
Q9NS98	SEMA3G	Semaphorin-3G	SEM3G_HUMAN	-4.329982593	0.04972163	0.009331839	0.068982418	32,623.98663	1622.117798	34,849.51072	7383.670112	33,439.52289	11,764.33253
Q15208	STK38	Serine/threonine-protein kinase 38	STK38_HUMAN	-4.314326762	0.050264137	0.043550666	0.16138934	17,720.98413	890.7299805	24,627.98869	7706.586399	26,995.66937	10,021.84456
Q9NT99	LRRC4B	Leucine-rich repeat-containing protein 4B	LRC4B_HUMAN	-4.152897004	0.056215158	0.003435788	0.042085303	49,791.42774	2799.032959	61,495.88143	11,835.26855	57,026.78345	13,349.67579
Q9BXF6	RAB11FIP5	Rab11 family-interacting protein 5	RFIP5_HUMAN	-4.067664878	0.059636323	0.002020711	0.032189078	49,783.66602	2968.914795	51,688.0699	10,924.91005	41,164.62188	13,449.93254
Q9Y534	CSDC2	Cold shock domain-containing protein C2	CSDC2_HUMAN	-3.95930705	0.064287985	0.000974334	0.021234095	59,826.00391	3846.093262	85,133.05957	9149.448948	79,482.06876	9484.767243
Q9UPQ8	DOLK	Dolichol kinase	DOLK_HUMAN	-3.915211269	0.066283277	0.002189497	0.033495736	14,577.39014	966.2371825	12,988.88131	3155.474141	9864.953951	3435.090327
Q15735	INPP5J	Phosphatidylinositol 4,5-bisphosphate 5-phosphatase A	PI5PA_HUMAN	-3.760654468	0.073778565	0.008338712	0.065647507	42,749.69629	3154.011231	51,893.4358	13,258.56071	48,934.18284	13,385.11016
Q03167	TGFBR3	Transforming growth factor beta receptor type 3	TGBR3_HUMAN	-3.716928682	0.076048907	6.41081E-05	0.003884262	74,017.58985	5628.956787	67,647.51123	9644.279839	45,067.71548	9364.680244
P01303	NPY	Pro-neuropeptide Y	NPY_HUMAN	-3.70910232	0.076462579	0.023746511	0.117709148	70,589.71485	5397.47168	265,627.7236	22,369.35032	402,567.9766	33,573.11999
P04271	S100B	Protein S100-B	S100B_HUMAN	-3.665845804	0.078789883	0.01199637	0.07934441	72,967.86964	5749.129885	131,072.7342	16,238.01367	167,118.8245	19,037.21353
Q8ND94	LRRN4CL	LRRN4 C-terminal-like protein	LRN4L_HUMAN	-3.556301974	0.085005384	9.22435E-06	0.001159193	178,939.7579	15,210.84278	179,886.3216	40,652.17763	43,391.23902	51,206.39809
P0DMM9	SULT1A3	Sulfotransferase 1A3;Sulfotransferase 1A4	ST1A3_HUMAN;ST1A4_HUMAN	-3.439001045	0.092205649	0.011010893	0.075431856	38,626.30176	3561.563233	44,209.27913	12,143.80417	37,863.4175	15,993.11712
P54760	EPHB4	Ephrin type-B receptor 4	EPHB4_HUMAN	-3.416078426	0.093682382	0.014001962	0.0868953	20,635.18402	1933.153198	31,146.54014	7027.536092	32,227.28658	10,137.11146
Q9BXJ2	C1QTNF7	Complement C1q tumor necrosis factor-related protein 7	C1QT7_HUMAN	-3.360765373	0.097343916	0.005995075	0.056991472	40,554.34863	3947.719116	39,863.91634	14,892.58891	17,333.73066	16,452.87469
Q9H9S4	CAB39L	Calcium-binding protein 39-like	CB39L_HUMAN	-3.352492875	0.097903695	0.006883947	0.060448077	29,382.13037	2876.619141	43,408.14957	9692.330892	42,781.72624	9657.160391
Q8WV41	SNX33	Sorting nexin-33	SNX33_HUMAN	-3.339444022	0.098793229	3.36148E-05	0.002563036	116,864.9258	11,545.46338	153,623.1849	26,463.49593	87,528.1971	27,439.50696

**Fig. 2:**
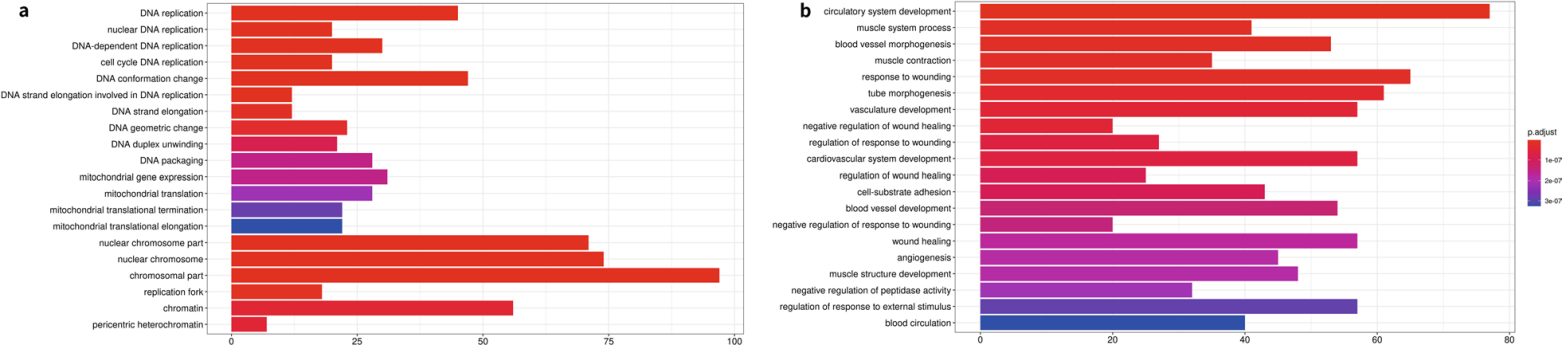
GO analysis of the up- and down-regulated proteins. (**a**) and (**b**) showed results of GO enrichment analysis for up- and down-regulated proteins respectively. The Y-axis represents the GO terms, and X-axis represents the Rich factor. Rich factor means the ratio of the number of DEPs to the total number of proteins annotated in this GO terms. The colour of the dot means different P.adjust value, and the size of dot indicates the number of DEPs in this term

To gain insights into the biological significance of these DEPs, the GO and KEGG enrichment analyses were performed through the R package (cluster Profiler, v3.16.1). As to the GO enrichment analysis for all these DEPs, the most enriched biological processes were DNA replication related, including nuclear DNA replication (GO:0,033,260), DNA replication (GO:0,006,260), cell cycle DNA replication (GO:0,044,786), and DNA strand elongation involved in DNA replication (GO:0,006,271), whereas the most enriched cellular components were the extracellular matrix (GO:0,031,012) and collagen-containing extracellular matrix (GO:0,062,023). Noteworthily, the GO term of the replication fork (GO:0,005,657) was also enriched in cellular components. Interestingly, most of the enriched GO terms in molecular functions were focused on DNA replication and the related energy metabolism, including DNA-dependent ATPase activity (GO:0,008,094), 3'-5' DNA helicase activity (GO:0,017,116), and others (Additional file 3: Table S3a). All these results indicated that DNA replication and cell proliferation associations were extremely frequent in SCCC. To get a more detailed view of SCCC, we further performed GO enrichment analysis for the up- and down-regulated DEPs separately. As for the up-regulated DEPs, all the enriched GO terms indicated the DNA replication and mitochondrial related in biological processes. In the molecular function, chromosomal part and mitochondrial envelope (cellular components), catalytic activity acting on DNA, and ATPase activity were mostly enriched (Fig. 2a). As for down-regulated DEPs, circulatory system development (biological processes), extracellular matrix (cellular components), and structural molecule activity (molecular function) were the most enriched terms (Fig. 2b).

**Fig. 3:**
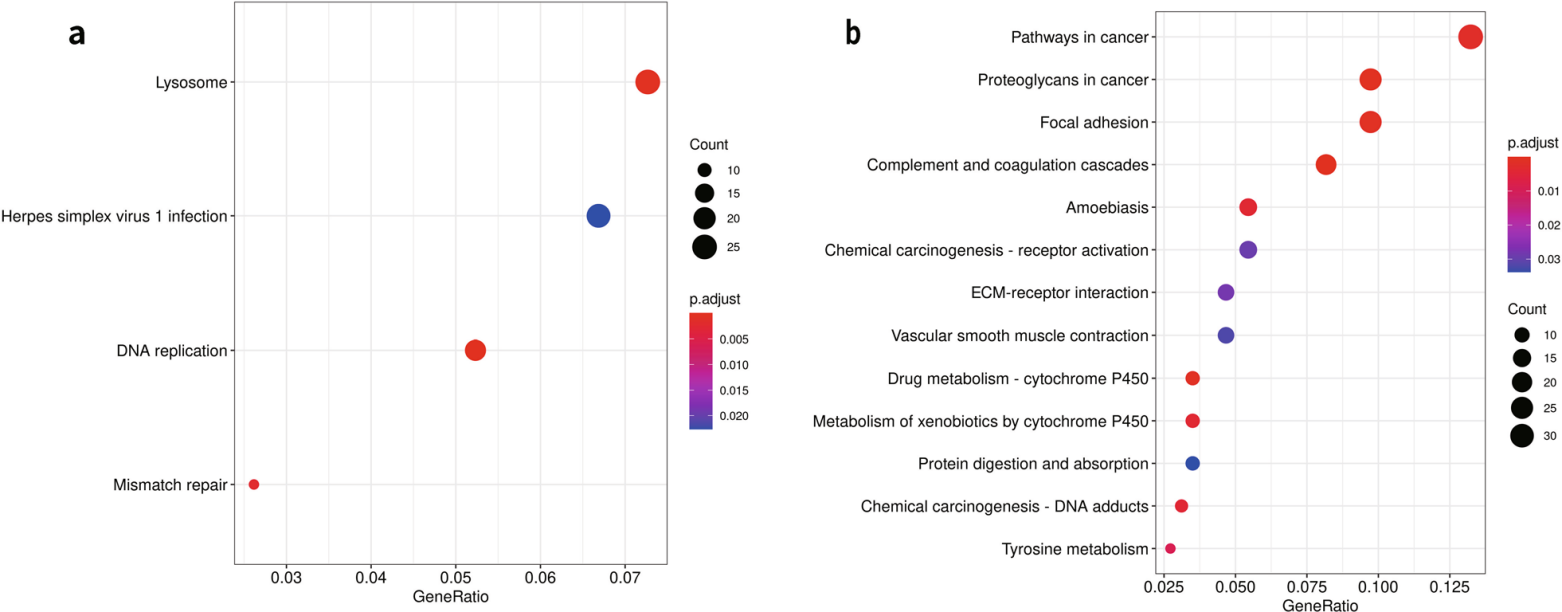
KEGG analysis of the up- and down-regulated proteins. (**a**) and (**b**) showed results of KEGG enrichment analysis for up- and down-regulated proteins respectively. The Y-axis represents the KEGG pathways, and X-axis represents the Rich factor. Rich factor means the ratio of the number of DEPs to the total number of proteins annotated in this pathway. The colour of the dot means different P.adjust value, and the size of dot indicates the number of DEPs in this pathway

Next, we performed a KEGG analysis to investigate key pathways involved in SCCC, which result in two pathways being significantly enriched, including the DNA replication (hsa03030) and Complement and coagulation cascades (hsa04610) (Additional file 3: Table S3b). Similarly, the up-and down-regulated DEPs were analyzed for KEGG enrichment. In the up-regulated group, the DEPs were enriched in the pathways of DNA replication (hsa03030), lysosome (hsa04142), mismatch repair (hsa03430), and Herpes simplex virus 1 infection (hsa05168) (Fig. 3a). For the down-regulated DEPs, the most enriched pathways were Complement and coagulation cascades (hsa04610), followed by Proteoglycans in cancer (hsa05205), Focal adhesion (hsa04510), and Drug metabolism-cytochrome P450 (hsa00982) (Fig. 3b).

### Validation of CDN2A and SYP expression via IHC

**Fig. 4:**
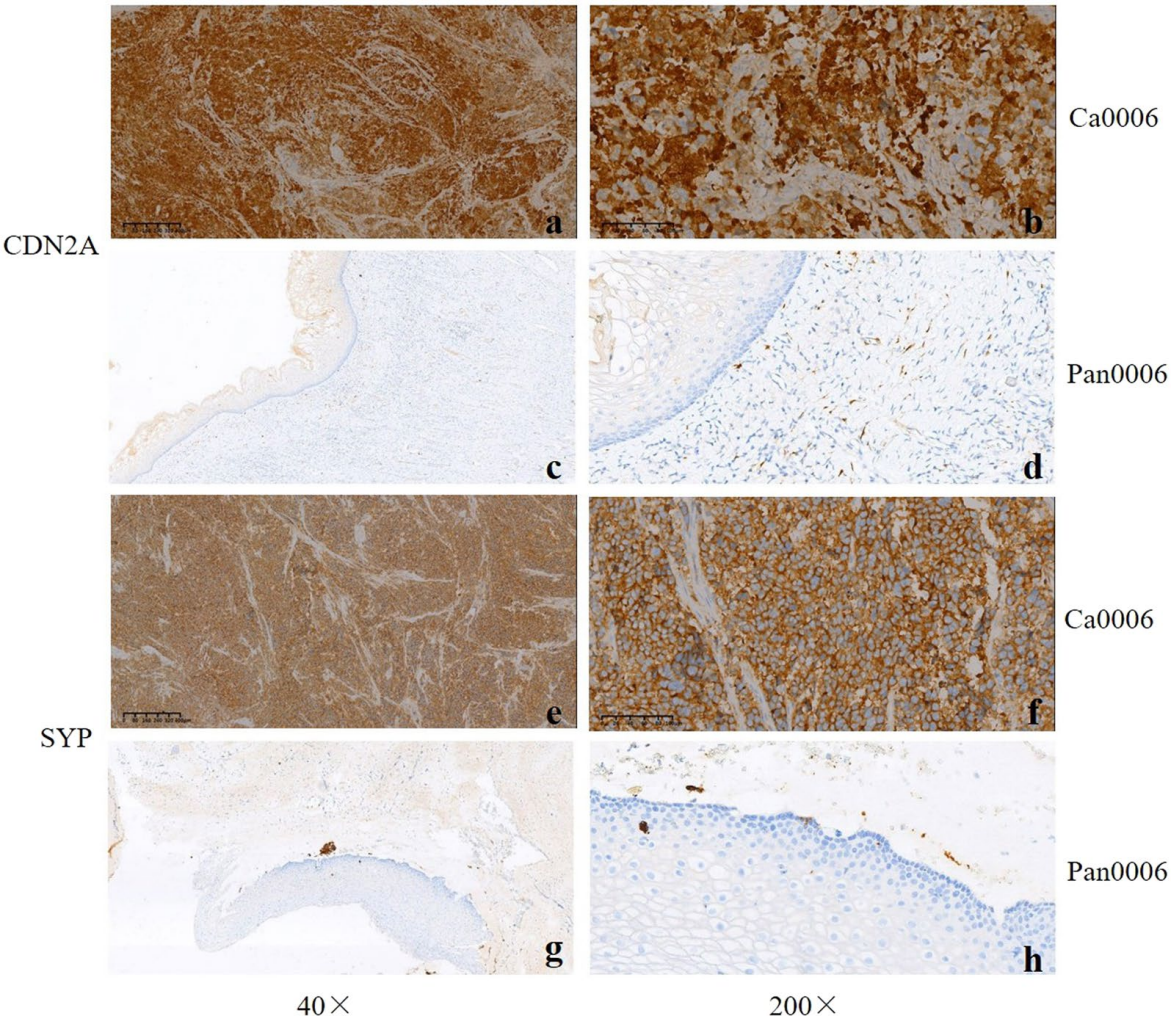
The representative staining results of CDN2A and SYP. **a**–**d** CDN2A was strongly positive in SCCC (case no. Ca0006) while negatively stained in the corresponding non-cancerous tissues; **e**–**h** SYP was positive Ca0006 but negative in Pan0006. The images were presented with 40 × and 200 × fields

**Fig. 5:**
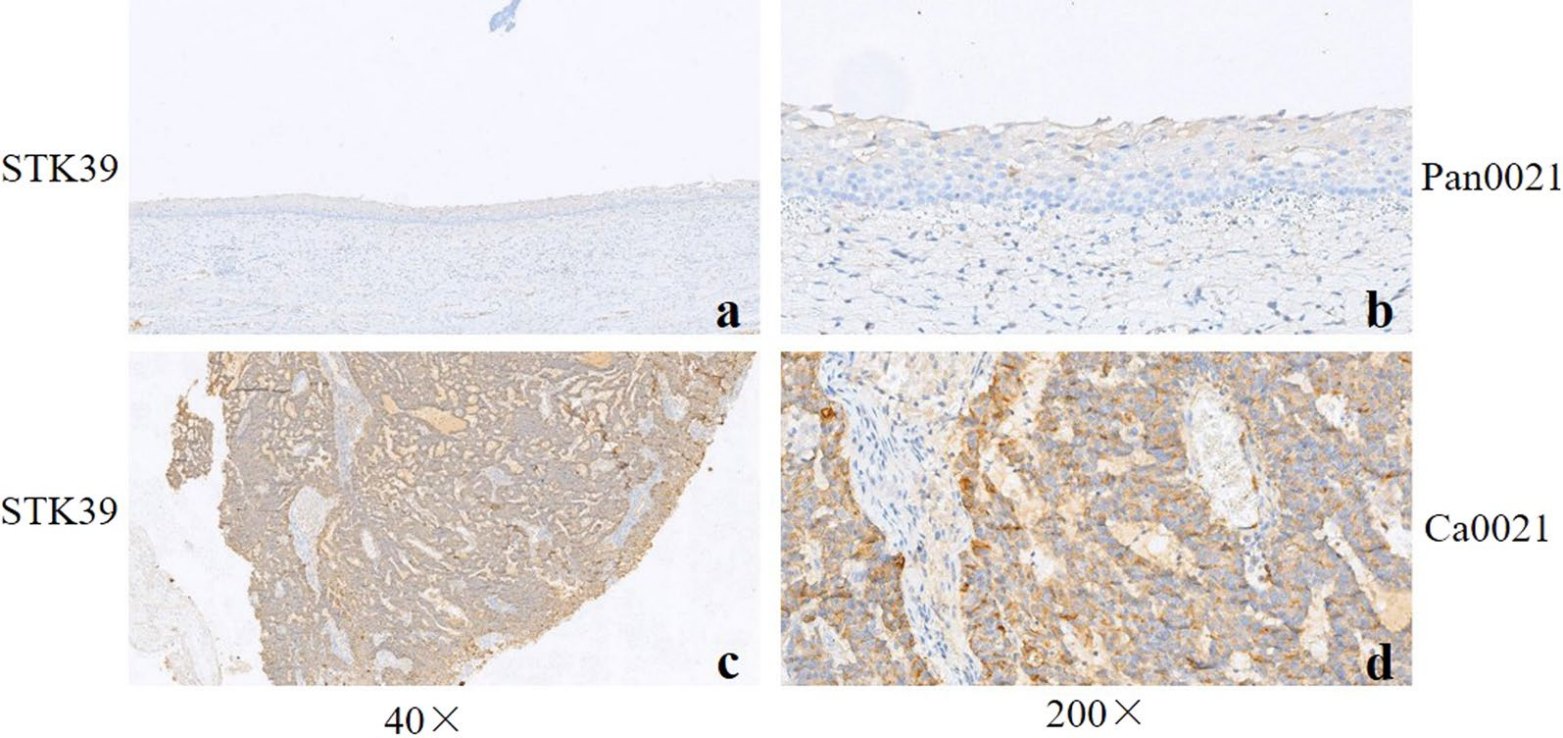
The representative images of STK39 staining. **a**, **b** The expression of STK39 was negative in normal tissues (case no. Pan0021); **c** and **d** STK39 was over-expressed in SCCC case (no. Ca0021). The images were presented with 40 × and 200 × fields

As previously reported, overexpression of CDN2A (Cyclin-dependent kinase inhibitor 2A, also known as P16, P42771) and SYP (Synaptophysin, P08247) was a distinctive feature of SCCC [13, 20, 21]. As shown by our data, the two proteins were both up-regulated in our cohort (CDN2A: rank 4, FC = 45.2, *P* = 0.001; SYP: rank 142, FC = 7.46, *P* = 0.011). For the validation, we investigated the protein level of CDN2A and SYP in all the SCCC and the matched non-cancerous tissues using IHC. Consistent with the proteomics results, CDN2A and SYP were positively stained in 88.9% (16/18) and 66.7% (12/18) SCCC tissues, while negatively stained in the non-cancerous samples (the representative figures were shown in Fig. 4). For further validation, we collected 5 more SCCC tissues to investigated the protein expression of STK39 (Top 1 of the up-regulated DEPs). The results of IHC demonstrated that STK39 was over-expressed in 80% (4/5) cases, while no expression of STK39 was detected in the adjacent normal cervix tissues (the representative figures were shown in Fig. 5).

### DEPs associated with recurrence in early stage SCCC

Surgery remained the first choice for SCCC at early stages, even though the recurrence rate was much higher than other types of cervical cancer. As for the seven-stage IB (2 IB1, 4 IB2, 1 IB3) cases of our study, five patients presented recurrence/metastasis and finally died of this disease; the DFS/OS were 21/31, 24/36, 17/20, 14/32, and 37/48 months. The two cases (both were stage IB2) with no recurrence were still alive until the last follow-up (OS were 62 and 67 months). To identify the proteins involved in SCCC recurrence, we further investigated DEPs between the two groups. At the cut-off (FC ≥ 1.5 and *P* < 0.05), 63 DEPs (24 up-regulated and 39 down-regulated) were identified (Table 4). Among the up-regulated proteins, BDH1 (Q02338) was the key enzyme for the catabolism of fatty acid, GNS (P15586) was involved in the catabolism of heparin/heparan sulfate/keratan sulfate, and ALDH9A1 (P49189) could catalyze the dehydrogenation of gamma-aminobutyraldehyde into gamma-aminobutyric acid (GABA). NAT1 (Q8WUY8) plays a key role in the catabolism of folate. NMNAT1 (Q9HAN9) and ATIC (P31939) are crucial for the biosynthesis of nicotinamide adenine dinucleotide and purine. Due to the limited sample size, the GO enrichment and KEGG pathway analyses were not performed.

**Table 4:** DEPs associated with tumor recurrence

Accession ID	Gene name	Protein descriptions	Protein names	FC_median	P value
P48668	KRT6C	Keratin, type II cytoskeletal 6C	K2C6C_HUMAN	0.072503932	0.024598382
Q01826	SATB1	DNA-binding protein SATB1	SATB1_HUMAN	0.124733095	0.044777843
P52732	KIF11	Kinesin-like protein KIF11	KIF11_HUMAN	0.148498322	0.015741424
Q5XPI4	RNF123	E3 ubiquitin-protein ligase RNF123	RN123_HUMAN	0.186961262	0.048216925
P49815	TSC2	Tuberin	TSC2_HUMAN	0.207848589	0.03074988
Q92994	BRF1	Transcription factor IIIB 90 kDa subunit	TF3B_HUMAN	0.216707696	0.021369094
Q5TBA9	FRY	Protein furry homolog	FRY_HUMAN	0.225588111	0.041696517
Q9GZX9	TWSG1	Twisted gastrulation protein homolog 1	TWSG1_HUMAN	0.22776201	0.047268744
P62328	TMSB4X	Thymosin beta-4	TYB4_HUMAN	0.243027058	0.037577203
Q5VSY0	GKAP1	G kinase-anchoring protein 1	GKAP1_HUMAN	0.252455748	0.000580324
Q96A46	SLC25A28	Mitoferrin-2	MFRN2_HUMAN	0.27202276	0.032086144
Q12926	ELAVL2	ELAV-like protein 2	ELAV2_HUMAN	0.298532229	0.03170129
P49069	CAMLG	Calcium signal-modulating cyclophilin ligand	CAMLG_HUMAN	0.302557561	0.000951223
Q9NX02	NLRP2	NACHT, LRR and PYD domains-containing protein 2	NALP2_HUMAN	0.303105419	0.043729258
Q9UH17	APOBEC3B	DNA dC- > dU-editing enzyme APOBEC-3B	ABC3B_HUMAN	0.307505559	0.042736708
Q0JRZ9	FCHO2	F-BAR domain only protein 2	FCHO2_HUMAN	0.31237225	0.017836496
Q8NHG7	SVIP	Small VCP/p97-interacting protein	SVIP_HUMAN	0.329904119	0.034044088
Q96EX3	WDR34	WD repeat-containing protein 34	WDR34_HUMAN	0.357116446	0.001481383
Q5VTL8	PRPF38B	Pre-mRNA-splicing factor 38B	PR38B_HUMAN	0.369479497	0.016564358
Q5SGD2	PPM1L	Protein phosphatase 1L	PPM1L_HUMAN	0.379674798	0.034282801
O95817	BAG3	BAG family molecular chaperone regulator 3	BAG3_HUMAN	0.381082841	0.042897533
O95873	C6orf47	Uncharacterized protein C6orf47	CF047_HUMAN	0.382205329	0.001378177
O14641	DVL2	Segment polarity protein dishevelled homolog DVL-2	DVL2_HUMAN	0.420672088	0.023989643
A6NCE7	MAP1LC3B2;MAP1LC3B	CCR4-NOT transcription complex subunit 1	CNOT1_HUMAN	0.422637802	0.03766099
O43169	CYB5B	Cytochrome b5 type B	CYB5B_HUMAN	0.44319756	0.038741469
Q01813	PFKP	ATP-dependent 6-phosphofructokinase, platelet type	PFKAP_HUMAN	0.445046286	0.030080123
O95379	TNFAIP8	Tumor necrosis factor alpha-induced protein 8	TFIP8_HUMAN	0.456239147	0.003659428
Q8WXF7	ATL1	Atlastin-1	ATLA1_HUMAN	0.4730972	0.001434618
P54920	NAPA	Alpha-soluble NSF attachment protein	SNAA_HUMAN	0.497840334	0.013526283
Q9NUU6	OTULINL	Inactive ubiquitin thioesterase OTULINL	OTULL_HUMAN	0.502225077	0.013731041
Q9NR31	SAR1A	GTP-binding protein SAR1a	SAR1A_HUMAN	0.507922855	0.021649547
Q9NRZ7	AGPAT3	1-acyl-sn-glycerol-3-phosphate acyltransferase gamma	PLCC_HUMAN	0.521302122	0.048957105
Q16537	PPP2R5E	Serine/threonine-protein phosphatase 2A 56 kDa regulatory subunit epsilon isoform	2A5E_HUMAN	0.538944117	0.043454481
P09497	CLTB	Clathrin light chain B	CLCB_HUMAN	0.55541691	0.021779062
O95721	SNAP29	Synaptosomal-associated protein 29	SNP29_HUMAN	0.600149351	0.043347253
O43264	ZW10	Centromere/kinetochore protein zw10 homolog	ZW10_HUMAN	0.609424832	0.002872554
P17612	PRKACA	cAMP-dependent protein kinase catalytic subunit alpha	KAPCA_HUMAN	0.63102747	0.022365444
P0DI81	TRAPPC2;TRAPPC2B	Paraneoplastic antigen-like protein 6A	PNM6A_HUMAN	0.635182531	0.020016126
O95359	TACC2	Transforming acidic coiled-coil-containing protein 2	TACC2_HUMAN	0.639384265	0.044236648
P17050	NAGA	Alpha-N-acetylgalactosaminidase	NAGAB_HUMAN	1.617067352	0.032694584
P31949	S100A11	Protein S100-A11	S10AB_HUMAN	1.705098227	0.040127176
Q0VGL1	LAMTOR4	Ragulator complex protein LAMTOR4	LTOR4_HUMAN	1.706522496	0.042665104
O95249	GOSR1	Golgi SNAP receptor complex member 1	GOSR1_HUMAN	1.711769645	0.014290968
P25686	DNAJB2	DnaJ homolog subfamily B member 2	DNJB2_HUMAN	1.726571422	0.03167421
P49189	ALDH9A1	4-trimethylaminobutyraldehyde dehydrogenase	AL9A1_HUMAN	1.823805109	0.008883657
P15586	GNS	N-acetylglucosamine-6-sulfatase	GNS_HUMAN	1.828353696	0.014993157
Q9NZQ3	NCKIPSD	NCK-interacting protein with SH3 domain	SPN90_HUMAN	1.848501531	0.048522891
Q92575	UBXN4	UBX domain-containing protein 4	UBXN4_HUMAN	1.874007659	0.010803221
Q6IPR1	ETFRF1	Electron transfer flavoprotein regulatory factor 1	ETFR1_HUMAN	1.899684535	0.026710264
P31939	ATIC	Bifunctional purine biosynthesis protein PURH	PUR9_HUMAN	1.917715158	0.043455261
Q8WWM9	CYGB	Cytoglobin	CYGB_HUMAN	1.943546861	0.010887103
P05543	SERPINA7	Thyroxine-binding globulin	THBG_HUMAN	2.029913652	0.037752149
Q86X76	NIT1	Deaminated glutathione amidase	NIT1_HUMAN	2.057725381	0.049984912
P18440	NAT1	Arylamine N-acetyltransferase 1	ARY1_HUMAN	2.294818464	0.037810236
Q9BS40	LXN	Latexin	LXN_HUMAN	2.373964585	0.01808862
O60911	CTSV	Cathepsin L2	CATL2_HUMAN	2.472069364	0.035432975
O75420	GIGYF1	GRB10-interacting GYF protein 1	GGYF1_HUMAN	2.523621756	0.043239593
Q9UNN8	PROCR	Endothelial protein C receptor	EPCR_HUMAN	2.697200689	0.038814284
Q53H47	SETMAR	Histone-lysine N-methyltransferase SETMAR	SETMR_HUMAN	2.702322143	0.039151164
Q9HAN9	NMNAT1	Nicotinamide/nicotinic acid mononucleotide adenylyltransferase 1	NMNA1_HUMAN	2.924298948	0.008769431
O75896	TUSC2	Tumor suppressor candidate 2	TUSC2_HUMAN	2.959401424	0.016305413
Q02338	BDH1	D-beta-hydroxybutyrate dehydrogenase, mitochondrial	BDH_HUMAN	4.07081439	0.042339021
Q8N465	D2HGDH	D-2-hydroxyglutarate dehydrogenase, mitochondrial	D2HDH_HUMAN	7.083717569	0.0431335

## Discussion

For SCCC, the significant aggressiveness and poor prognosis (even after extensive treatments) have been well documented in previous studies [22]. This could be mainly attributed to the native behaviors of SCCC tumors, such as rapid proliferation and innate resistance to current therapies [22]. Several previous studies have tried to explore the genetic aberrations in SCCC. The common events were LOH at chromosome 9p and 3p and TP53 mutations, which also occurred frequently in a wide range of human malignancies. Using the whole-exome sequencing, mutations of key genes of PI3K/AKT/mTOR were also detected in several SCCC cases. Compared with the findings at DNA and RNA levels, there was limited knowledge about unique protein profiles in SCCC. In our study, both GO enrichment and KEGG pathway analysis showed that the up-regulated DEPs were significantly correlated with DNA replication, chromosome duplication, allocation, and conformation change, indicating the vigorous mitosis of SCCC cancer cells. This is crucial for the extraordinary growth of SCCC tumors, especially under the pressure of radiation and chemotherapy [23]. Besides genetic materials, the abundant energy supply was inevitable for the rapid tumor growth [23]. Thus, it was not surprising to find that the most enriched molecular functions were ATPase activity, catalytic activity acting on DNA, and DNA helicase activity. Collectively, the above findings demonstrated that uncontrolled proliferation was a distinctive feature of SCCC and these abnormal proteins and pathways should be considered as potential targets for developing novel therapies.

Besides CDN2A (Cyclin-dependent kinase inhibitor 2A, also known as P16), other significantly upregulated DEPs were also candidate markers for SCCC diagnosis and treatment, including STK39 (a STE20/SPS1-related proline-alanine-rich protein kinase), ZMYM2 (Zinc finger MYM-type protein 2), CKMT1A (Creatine kinase U-type, mitochondrial), and HIP1R (Huntingtin-interacting protein 1-related protein). As previously reported, STK39 was involved in the development and progression of various human malignancies. In lung carcinoma, the over-expression of STK39 was associated with advanced tumor stage and poor prognosis [24]. Similar findings were also detected in human osteosarcoma and hepatocellular carcinoma [25, 26]. Mechanistically, it was revealed that STK39 bound with PLK1 and then activated MAPK signaling pathway, which consequently promoted tumor proliferation and aggression in hepatocellular carcinoma [25]. Furthermore, in cervical cancer, STK39 significantly enhanced tumor invasion via activating the NF-κB/p38-MAPK/MMP2 signaling pathway [27]. Consistently, our protein–protein interaction analysis between STK39, MAPT and MAPKs (including MAPK1, MAPK13 and MAP2K1). In our validation cohort, the over-expression of STK39 was detected in 80% SCCC cases, while in none of the normal control tissues. This might indicate a potential role of STK39 in SCCC, which attracted our attention for further explorations.

As our results shown, most up-regulated DEPs were associated with DNA replication, especially the nuclear DNA replication. ZMYM2 was one of them and ranked as the second mostly up-regulated DEP. The ZMYM2 protein was a zinc finger protein which participate into a histone deacetylase complex which was activated in many kinds of cancers to inhibit the functions of tumor suppressor genes [28, 29]. The latest findings proved that ZMYM2 could constrain 53BP1 from binding chromatin and thus promote the DSB (double-strand break) repair in a BRCA-dependent manner [30]. The overexpression of ZMYM2 was also detected in human ovarian cancer, which could remarkedly promote tumor growth in vitro and in vivo [31]. Furthermore, destruction of the ZMYM2-containing complex was proposed as a therapeutic strategy to overcome the stemness of ovarian cancer cells [31].

According to the current knowledge, the role of HIP1R remains controversial. In gastric cancer, HIP1R inhibited the AKT pathway and served as a tumor suppressor via promoting apoptosis and inhibiting tumor invasion [32]. On the contrast, Burnstein et al. proved that HIP1R was significantly upregulated in metastatic prostate cancer [33]. In vitro, HIP1R functioned as an oncogene to enhance the invasion and migration of human prostate cancer cells [33]. Consistently, in non-small cell lung carcinoma, patients with higher expression of HIP1R presented worse progression-free survival and overall survival than those with lower HIP1R [34]. Interestingly, the authors found that HIP1R was negatively correlated with PD-L1 level and served as an independent predictor for tumor response to the anti-PD-1 treatment [34]. Moreover, Xu et al. recently proved that HIP1R could bind PD-L1 at a conserved domain and then deliver PD-L1 to the lysosome for proteolysis. Thus, tumor cells with high expression of HIP1R presented lower PD-L1 level and poor response to the therapy targeting PD-1/PD-L1 signal [35]. As previously reported, PD-L1 was notably lower in SCCC than those in either squamous cell cancer or adenocarcinoma [36, 37]. In our current study, PD-L1 was excluded for analysis due to its extreme low abundance, which did not provide a direct correlation between high level of HIP1R and low level of PD-L1 and needed further investigation.

Compared to its low incidence at other sites, more than 95% of small cell carcinomas arise in the lung (SCLC), accounting for 15–20% of all lung cancer [38]. Therefore, most of the previous research findings of small cell carcinomas were obtained in SCLC. And unsurprisingly, most of the current treating strategies for small cell carcinoma were also learned from those for SCLC [39]. In another study, the authors found that SCCC and SCLC shared similar protein expression profiles, while different from those of squamous cell cancer or adenocarcinoma of the cervix. Although the sample size is quite small, 16 up-regulated proteins in SCCC were identified [40]. Interestingly, *NT5DC2* and *VRK1* were also up-regulated in the SCCC cases of our cohort. In the contrast, Schultheis et al. demonstrated that SCCC harbored a low mutation burden, few copy number alterations, and other than *TP53* in two cases (2/9, 22.2%) no recurrently mutated genes. The majority of mutations were likely passenger missense mutations and only a few affected previously described cancer-related genes [41]. By screening the public database, they also concluded that the overall non-silent mutation rate of SCCC was significantly lower than that of SCLC, HPV-driven cervical adenoma- and squamous cell carcinomas, or HPV-positive head and neck squamous cell carcinomas [41]. These findings indicated that SCCC might be of unique molecular characteristics and more research is warranted to uncover the details.

Treated with surgery and appropriate adjuvant therapies, the prognosis for early-stage SCCC was relatively better than for advanced tumors [7]. However, tumor relapse and metastasis were not rare in this group. In our cohort, a different group of DEPs was identified to be associated with tumor recurrence. Their functions were associated with catabolism of fatty acid (BDH1), heparin/heparan sulfate/keratan sulfate (GNS) and gamma-aminobutyric acid (ALDH9A1), folate (NAT1), and biosynthesis of nicotinamide adenine dinucleotide (NMNAT1), purine (ATIC) [42–45]. Importantly, these products were the necessary raw material for DNA replication and mitosis. Moreover, NAT1 also could help metabolize drugs and other xenobiotics, which might be accountable for the frequent chemo-resistance in SCCC [46]. Collectively, our findings might provide novel clues for the surveillance and prognosis prediction in SCCC patients.

Angiogenesis is inevitable for the multi-stage development of most cancers, as it ensures the supply pipe to transport nutrients and oxygen into tumor mass while removing the metabolic waste [47]. Therefore, anti-angiogenesis drugs could notably suppress tumor growth and present synergistic effects when combined with other treatments [48]. Unexpectedly, in the present study, a group of proteins related to the development of the circulatory system was downregulated, implying a relatively lower activity of neovascularization in SCCC. A similar phenomenon was reported in the high-aggressive pancreatic ductal adenocarcinomas, which was largely avascular and not sensitive to anti-angiogenic regimens [47, 49]. In another retrospective study, we reviewed 24 recurrent and/or metastasized SCCC who received anti-angiogenic drugs. According to the preliminary results, most cases were not sensitive to either the specific competing antibody of VEGF (Bevacizumab) or small molecules targeting VEGFRs (Anlotinib and Apatinib) (not published yet). The poor response rate might be partially attributed to the low level of angiogenesis in SCCC, which was planned to be validated in our further study.

## Conclusions

In conclusion, we first reported the protein expression signature of SCCC using quantitative proteomics analysis. Moreover, a panel of key proteins (enzymes) was shown to be associated with SCCC recurrence. Their functions were mainly related to the catabolism or biosynthesis of indispensable substances for DNA replication and organelle formation. These findings revealed novel targets for treating SCCC in the future.

## Supplementary Information

**Additional file 1. **Additional Table, supplementary Table 1, overview of the proteomics profiles identified by DIA in SCCC and normal cervix tissues.

**Additional file 2. **Additional Table, supplementary Table 2, the differentially expressed proteins (DEPs) associated with SCCC.

**Additional file 3.** Additional Table, supplementary Table 3, functional enrichment analysis of DEPs associated with SCCC. (**a**) GO enrichment of DEPs between SCCC and normal cervix tissues. (**b**) KEGG enrichment of DEPs between SCCC and normal cervix tissues.

**Additional file 4.** Additional Table, supplementary Table 4, the qualitative differences between SCCC and normal tissues.

## Data Availability

All data are supplied in the article or as the additional materials.
